# Adherence predictor variables in AIDS patients: an empirical study using the data mining-based RFM model

**DOI:** 10.1186/s12981-020-00326-8

**Published:** 2021-01-28

**Authors:** Min Li, Qunwei Wang, Yinzhong Shen

**Affiliations:** 1 Shanghai Public Health Clinical Center, Fudan University, Shanghai, 201508 China; 2 College of Economics and Management, Nanjing University of Aeronautics and Astronautics, Nanjing, 211106 Jiangsu China

**Keywords:** Antiretroviral therapy, Adherence prediction, RFM, AIDS

## Abstract

**Background:**

Highly active antiretroviral therapy (ART) is still the only effective method to stop the disease progression in acquired immunodeficiency syndrome (AIDS) patients. However, poor adherence to the therapy makes it ineffective. In this work, we construct an adherence prediction model of AIDS patients using the classical recency, frequency and monetary value (RFM) model in the data mining-based customer relationship management model to obtain adherence predictor variables.

**Methods:**

We cleaned 257,305 diagnostic data elements of AIDS outpatients in Shanghai from August 2009 to December 2019 to obtain 16,440 elements. We tested the RFM and RFm (R: recent consultation month, F: consultation frequency, M/m: total/average medical costs per visit) models, three clustering methods (K-means, Kohonen and two-step clustering) and four decision algorithms (C5.0, the classification and regression tree, Chi-square Automatic Interaction Detector and Quick, Unbiased, Efficient, Statistical Tree) to select the optimal combination. The optimal model and clustering analysis were used to divide the patients into two groups (good and poor adherence), then the optimal decision algorithm was used to construct the prediction model of adherence and obtain its predictor variables.

**Results:**

The results revealed that the RFm model, K-means clustering analysis and C5.0 algorithm were optimal. After three rounds of k-means clustering analysis, the optimal RFm clustering model quality was 0.8, 10,614 elements were obtained, including 9803 and 811 from patients with good or poor adherence, respectively, and five types of patients were identified. The prediction model had an accuracy of 100% with the recent consultation month as an important adherence predictor variable.

**Conclusions:**

This work presented a prediction model for medication adherence in AIDS patients at the designated AIDS center in Shanghai, using the RFm model and the k-means and C5.0 algorithms. The model can be expanded to include patients from other centers in China and worldwide.

## Introduction

Acquired immunodeficiency syndrome (AIDS), caused by the human immunodeficiency virus (HIV), is one of the most serious infectious diseases in the world [1], with no cure or effective vaccine so far. Highly active antiretroviral therapy (ART), developed in 1996, is still the only effective method to inhibit HIV replication. This method changed AIDS from a lethal disease to a treatable chronic infectious disease [2–4]. Furthermore, it can rebuild the immune system in AIDS patients to prevent death caused by various opportunistic infections [3, 5]. However, the effectiveness of this treatment depends on the patients’ adherence to the therapy [3]. High adherence can reduce drug-resistant strains and further transmission of such strains and decrease the AIDS-related opportunistic infections, mortality rate, incidence, treatment costs and disease burden [3, 6–8].

In 2003, the World Health Organization defined adherence as the extent to which a person’s medication-taking behavior, dietary compliance and/or execution of lifestyle changes correspond with the agreed recommendations from a healthcare provider in the disease treatment and control. Basically, it is the degree to which a patient sticks to a treatment plan [9, 10]. Poor adherence is a widespread problem in the world [1].

The minimum required adherence level to achieve the disease inhibition was shown to be 90% by Chen [11]. The same indication was also presented by Kioko [12], Mbengue [13] and Neupane [14]. However, according to the current studies, adherence is poor in AIDS patients. Mbengue [13] found that only 26.67% of the patients exhibit high adherence, Neupane [14] found that only 87.4% of the patients have good adherence, Souza (2019) [15] found that only 52.5% of the patients have good adherence, whereas 33.3% of the patients have low adherence. Sagarduy [16] found that only 82% of the patients are adherent to ART. Poor adherence might be caused by the lack of an objective, simple and easily understood operational marker for determining adherence. Therefore, developing a marker to monitor the clinical treatment and evaluate adherence in AIDS patients will help to give more time and attention to the patients with low adherence to improve it and increase the therapy effectiveness.

Currently, the following methods are used in China and other countries to determine adherence in AIDS patients: (1) self-reported evaluation questionnaires [9, 11, 13, 14, 16–20], (2) tablet, prescription and pharmacy records for drug count [21, 22], (3) plasma drug concentration monitoring [10, 22, 23] and (4) a mixture of self-reported questionnaires and plasma drug concentration monitoring. However, method (1) tends to be limited by patient-related subjective factors, and its reliability and validity tend to be questionable. Method (2) requires the inspector to have high professional knowledge, and electronic pillboxes are expensive and difficult to promote. As for method (3), it is limited by shortcomings such as the difficulty of collecting blood every day, high testing costs and large differences in the metabolism of individuals.

Given the drawbacks in the currently used measurement methods, Haberer [24] indicated the need to improve the adherence measurement in resource-limited settings to improve the ART adherence on a large scale. Thus, there were some attempts to build adherence prediction models. Krumme [25] used the classical recency, frequency and monetary value (RFM) model [26] in the customer relationship management (CRM) theory [26–28] to predict that the adherence of the patients buying cardiovascular drugs from retail pharmacies was positively correlated with the number of store visits per month and the dollar amount per visit. Zare Hosseini [26] employed clustering analysis and decision algorithms in an RFM model-based data mining for the prediction using the data of patients in Iran and found that the adherence in hospital patients is associated with the recent consultation time, hospital treatment cycle, consultation frequency and total amount paid. This reveals that the RFM model is effective in predicting the variables for chronic disease medication adherence.

In this study, we used the classical RFM model in the data mining-based CRM theory [26–28] to obtain predictor variables for adherence in AIDS patients [25, 26, 29]. The presented model shows an efficiency to guide improvements in the adherence in AIDS patients.

## Materials and methods

### Research design

This is an empirical study performed on the data exported from the hospital information system (HIS) of the only designated hospital in Shanghai for treating AIDS patients from August 2009 to December 2019. The data was used to train and test an RFM model to get predictor variables for adherence in AIDS patients.

### Data extraction and preprocessing

The data of AIDS outpatients from August 1, 2009, to December 31, 2019 were exported from the HIS of the research unit using the methods from literature [25, 26, 29, 30]. The fields included the consultation time, patient’s identification card number, gender, age, place of residence (local/no-local) and medical costs, for a total of 257,305 data elements (16,440 patients). Public hospitals in Shanghai in China implement a system wherein the actual name of the patient is used during consultation, and the identification number of the patient is an essential field. The SPSS 22.0 and SPSS Modeler 18.0 software were used for data analysis.

The data were cleaned, and the following fields were expanded by the methods used in literature [25, 26, 30]: (1) The consultation time field was expanded to “recent consultation month”, with December 2019 as the first month, November 2019 as the second month and so on until August 2009 as the 125th month; (2) The cumulative cost field in the patient’s identification card was used to calculate the “total medical costs” field; (3) The consultation time and cumulative frequency in the patient’s identification card were used to obtain the “consultation frequency” field; (4) The “total medical costs” field of each patient was divided by the “consultation frequency” field to obtain the “average medical costs per visit”. These constitute one data element representing one person.

### Variable generation and descriptive statistical analysis

Seven variables (recent consultation month, gender, age, total medical costs, consultation frequency, average medical costs per visit and place of residence) were generated. The factors of gender, age and place of residence were statistically analyzed to investigate good or poor acceptability, then four variables (recent consultation month, total medical costs, consultation frequency and average medical costs per visit) were used to describe these 16,440 data elements as mentioned in literature [25, 26, 29, 30].

### Finding the optimal RFM or RFm model, clustering analysis and decision algorithm

In this experiment, we tested the RFM and RFm models with several clustering analysis and decision algorithms to determine the best components to construct and evaluate the adherence prediction model. We employed methods from literature [25, 26, 29] and used the RFM model theory as follows: (1) The three fields of recent consultation month, consultation frequency and total medical costs were used for the RFM model [26]; (2) The three fields of recent consultation month, consultation frequency and average medical costs per visit were used for the RFm model [25, 29]. Three clustering methods (K-means, Kohonen and two-step clustering) were used to construct the clustering models, in which four decision algorithms (C5.0, classification and regression tree (CART), Chi-square Automatic Interaction Detector (CHAID) and Quick, Unbiased, Efficient, Statistical Tree (QUEST)) were used in each model to construct several preliminary prediction models. From these models, we determined the optimal RFM(m) model, clustering analysis method and decision algorithm based on the quality of the model and stability of important predictor variables, which were used for the adherence prediction model experiment.

### Validating the adherence prediction model and obtaining the variables

In this experiment, we used the optimal RFM(m) model and methods found in the previous experiment to construct the best clustering model, separate patients with good adherence from those with poor adherence and identify important variables for adherence prediction. The literature methods were used as references, and the optimal decision algorithm was employed, with good and poor adherence as targets. The important predictor variables in the best clustering model were utilized as the input variables, and the data underwent randomization and binning: we used 90% of the data as the training set to construct the adherence prediction model, and the remaining 10% was used as the test set to validate the model and finally obtain the adherence predictor variables.

## Results

### Descriptive statistical analysis of preprocessed data

The data preprocessing resulted in 16,440 data elements, such that each element represented one patient. The mean recent consultation month of the 16,440 AIDS patients was 15 months, and the median was 3 months. The mean total medical cost was 11,000 RMB (China’s currency), and the median was 8700 RMB. The average medical cost per visit was 900 RMB, and the median was 644 RMB. The mean consultation frequency was 15.65 visits, and the median was 13 visits. These four markers conform to a positively skewed distribution. The results are shown in Table 1. Since there was no statistically significant difference between good and bad adherence according to the factors of gender, place of residence and age (see Table 2), only the descriptive statistical analysis was conducted.

**Table 1:** Consultation status of AIDS patients in 2009–2019

Content	Samples	Minimum (M)	Maximum (X)	Mean (E)	Standard deviation	Median	Skewness	Kurtosis
Recent consultation month (month)	16,440	1	125	14.99	23.58	3	2.111	4.24
Total medical cost (RMB)	16,440	1	666,737.98	11,315.29	17,503.54	8740.51	16.88	496.76
Average medical cost per visit (RMB)	16,440	1	28,270	918.01	1107.12	644.47	4.98	57.29
Consultation frequency (visits)	16,440	1	397	15.65	14.90	13	3.58	52.43

**Table 2:** Cross-tab analysis of adherence with gender, residence and age

Content	Variable	Good acceptability	Poor acceptability	Total
Gender	Male	8934	738	9672
	Female	869	73	942
	Total	9803	811	10,614
Place of residence	Local	6962	536	7498
	No-local	2841	275	3116
	Total	9803	811	10,614
Age	≤ 5 years old	1	0	1
	6–11 years old	2	0	2
	12–15 years old	2	2	4
	16–18 years old	10	2	12
	19–22 years old	163	11	174
	23–29 years old	1632	149	1781
	30–39 years old	3368	312	3680
	40–49 years old	1758	136	1894
	50–59 years old	1449	101	1550
	60–69 years old	1070	70	1140
	70–79 years old	308	22	330
	80–89 years old	39	6	45
	≥ 90 years old	1	0	1
	Total	9803	811	10,614

### The optimal RFM(m) model, clustering analysis and decision algorithm

The three markers in the RFM model were used as variables to construct 13 models (including five clustering models and eight prediction models). However, the predictor variables were unstable. The three markers in the RFm model were then used as variables to construct 27 models (including 7 clustering models and 20 prediction models), the predictor variables were stable, and the model was robust. Clustering analysis was used to construct 12 models, and the k-means clustering analysis was the most robust one. The decision algorithm was used to construct 28 models, and the C5.0 algorithm was robust and had high prediction accuracy. The results showed that the RFm model, k-means clustering analysis and C5.0 algorithm were optimal. The results are shown in Table 3.

**Table 3:** Preliminary experiment on RFM(m) models, clustering analysis, and decision algorithms in 16,440 valid datasets obtained after cleaning

Model type	Clustering analysis				Decision algorithm							
	Clustering type	Number of models constructed	Model quality	Predictor variable importance	Predictor variable importance				Prediction model accuracy (%)			
					C5.0	CHAID	CART	QUEST	C5.0^4^	CHAID	CART	QUEST
RFM model^1^	K-means	1st round	0.8	R = 1 M = 1 F = 1	R = 0.9865 M = 0.0067 F = 0.0067	R = 0.7402 M = 0.2547 F = 0.0052	R = 0.9697 M = 0.0152 F = 0.0152	R = 0.9689 M = 0.0156 F = 0.0156	99.96	93.55	99.77	97.07
		2nd round	0.9	R = 1 M = 1 F = 1	M = 1	M = 0.7039 F = 0.2961	_	_	99.98	99.9	_	_
		3rd round	0.7	R = 1 M = 1 F = 1	M = 0.5 F = 0.5	M = 1	_	_	99.98	99.93	_	_
	Two-step clustering	1st round	0.4	R = 1 M = 1 F = 1	_	_	_	_	_	_	_	_
	Kohonen	1st round	0.4	R = 1 M = 1 F = 1	_	_	_	_	_	_	_	_
RFm model^2^	K-means^3^	1st round	0.5	R = 1 m = 1 F = 1	R = 0.6735 m = 0.017 F = 0.3095	R = 0.6661 m = 0.0429 F = 0.2910	R = 0.7172 m = 0.0020 F = 0.2808	R = 0.7479 m = 0.0017 F = 0.2503	99.88	90.38	97.43	95.86
		2nd round	0.8	R = 1 m = 1 F = 1	R = 0.5918 m = 0.4043 F = 0.0039	R = 0.5938 m = 0.1768 F = 0.2293	R = 0.5814 m = 0.4129 F = 0.0057	R = 0.5962 m = 0.3999 F = 0.0039	99.96	96.66	98.06	98.33
		3rd round	0.6	R = 1 m = 1 F = 0.37	R = 0.7258 m = 0.0451 F = 0.1841	R = 0.7708 m = 0.2728 F = 0.0014	R = 0.971 m = 0.0268 F = 0.0022	R = 0.6749 m = 0.3245 F = 0.0007	99.82	97.48	97.48	98.25
	Two-step clustering^5^	1st round	0.7	R = 1 m = 1 F = 1	R = 0.6864 m = 0.2090 F = 0.1046	R = 0.6607 m = 0.2962 F = 0.0431	R = 0.6283 m = 0.2582 F = 0.1135	R = 0.5797 m = 0.2624 F = 0.1579	99.87	96.9	98.41	97.32
		2nd round	0.7	R = 1 m = 0.15 F = 0.01	R = 0.7058 m = 0.2942	R = 0.8428 m = 0.1572	R = 0.7351 m = 0.2649	R = 0.7398 m = 0.2602	98.61	95.45	98.04	97.7
		3rd round	0.4	R = 1 m = 1 F = 1	_	_	_	_	_	_	_	_
	Kohonen	1st round	0.4	R = 1 m = 1 F = 1	_	_	_	_	_	_	_	_

R: Recency; F:Frequency; M: Monetary. Values lie in the 0–1 range

^1^The predictor variables of the RFM model were either unstable, or could not be used for modeling in clustering analysis and decision tree algorithm. M: total medical costs

^2^The predictor variables of the RFm model in the decision algorithm were stable. m: average medical costs per visit

^3^The K-means clustering model was robust

^4^The C5.0 algorithm prediction model had an accuracy of 99%

^5^The accuracy of the two-step clustering in the C5.0 algorithm was lower than that of the k-means clustering model, and the quality of the model in the third round was low

### The adherence prediction model and variables

The R, F and m in the RFm model were used as the variables for three rounds of k-means clustering analysis before the C5.0 algorithm was used to construct and validate the adherence prediction model. The 16,440 data elements underwent one round of k-means clustering analysis to remove the data of the patients who did visit within 24 months. The second round removed the data of the patients who did not visit within 8 months. The third round resulted in the best model quality of 0.8, and 5 clusters representing 5 types of patients. Among these elements, 9803 (recent consultation month ≤ 3 months) were patients with good adherence, and 811 (recent consultation month > 3 months) were patients with poor adherence. Furthermore, two important predictor variables (recent consultation month and average medical costs per visit) were obtained. The results are shown in Tables 4 and Table 5.

**Table 4:** Results of cleaned 16,440 datasets after three rounds of K-means clustering analysis

Samples	Number of constructed models	Clustering number (type)	Model quality	Predictor variable importance
16,440	1st round	6	0.5	R = 1 m = 1 F = 1
11,585	2nd round	6	0.7	R = 1 m = 1 F = 1
10,614	3rd round	5	0.8	R = 1 m = 1 F = 0.0563

R: Recency; F: Frequency; m: average medical costs per visit. Values lie in the 0–1 range

**Table 5:** Clustering map of five types of patients (10,614 datasets) after three rounds of k-means clustering analysis

Cluster	Cluster-4	Cluster-1	Cluster-3	Cluster-2	Cluster-5
Label	Good adherence	Good adherence	Good adherence	Poor adherence	Poor adherence
Sample (Patient ratio %)	4313 (40.6%)	2966 (27.9%)	2406 (22.7%)	811 (7.6%)	118 (1.1%)
input	Average medical costs per visit = 651.76	Average medical costs per visit = 678.68	Average medical costs per visit = 682.59	Average medical costs per visit = 831.28	Average medical costs per visit = 3733.77
	Recency = 1.00	Recency = 2.00	Recency = 3.00	Recency = 4.62	Recency = 1.47
	Frequency = 20.75	Frequency = 21.63	Frequency = 20.93	Frequency = 16.36	Frequency = 16.67

Since the AIDS patients in the study unit can collect drugs for free from the designated hospital once every 3 months, and following the recommendation of Tarokh [30] to designate 3 months as one consultation cycle, we decided to classify the patients as well adherent if the recent consultation month was between one and three months. As a result, the patients in Clusters 1, 3, 4 and 5 were classified as patients with good adherence. The patients in Cluster 5 had other underlying diseases. Therefore, the average medical costs per visit was relatively high. The patients in Cluster 2, who did not go for consultation for more than 4 months, were classified as poorly adherent. The C5.0 algorithm was employed, with good adherence and poor adherence as the targets, and the recent consultation month and average medical cost per visit as the input variables. Validating the adherence prediction model showed that the recent consultation month represented the adherence prediction model node. If recency ≤ 3, Model 1 has a good adherence. If recency > 3, Model 2 has a poor adherence. Thus, there was only one important predictor variable: the recent consultation month. The accuracy of the prediction model was 100%. The results are shown in Table 6.

**Table 6:** C5.0 algorithm analysis results

Item	Data binning	Node/model^1^	Predictor variable importance	Prediction model accuracy (%)			Amount of data (sets) indicating good/poor adherence		
Training set	90% data	R ≤ 3/[Model: 1] R > 3/[Model: 2]	R = 1	Correct	9582	100%	Good adherence	8846	0
				Wrong	0	0	Poor adherence	0	736
				Total	9582	–	Total	9582	
Test set	10% data	R ≤ 3/[Model: 1] R > 3/[Model: 2]	R = 1	Correct	1032	100%	Good adherence	957	0
				Wrong	0	0	Poor adherence	0	75
				Total	1032	–	Total	1074	

90% of the data was used as the training set to construct the adherence prediction model, and the remaining 10% was used as the test set to validate the model

^1^Nodes: Recency: R; 3 months was used as a node to divide R into two categories: for Model 1, R ≤ 3 months; indicating good adherence; for Model 2, R > 3 months, indicating that poor adherence

## Discussion

In this study, we used 257,305 consultation data elements that were generated for 16,440 AIDS patients in 125 months (August 1, 2009 to December 31, 2019) to train and test an adherence prediction model.

In the preliminary experiment, we referenced the classical RFM model [26, 29] that is used in the CRM theory in various industries. Zare Hosseini [26] used the total medical costs (RFM model), whereas Krumme [25] used the average cost per visit (RFm) model for the analysis. Aiming to have an overall assessment of the RFM/m models and different clustering analysis and decision algorithms, we constructed RFM and RFm models, and evaluated the predictor variable stability and clustering model robustness to find the best elements. The results showed that the RFm model was superior to the RFM model (Table 3). Furthermore, the k-means and two-step clustering models had good performance. However, in the C5.0 algorithm, the model prediction accuracy values of the first two rounds of the two-step clustering were slightly lower than that of the k-means clustering analysis (Table 3), and its clustering quality in the third round was only 0.4. Therefore, the k-means clustering was shown to be better than the other tested clustering methods. Compared with the CART, CHAID and QUEST algorithms, the C5.0 algorithm model was robust and had a prediction accuracy of 99%. Hence, it represented the best prediction model (Table 3).

In the adherence prediction model experiment, k-means clustering analysis was employed for three rounds of clustering on 16,440 valid data elements to obtain a good clustering model and to achieve normal data distribution, since the variables were not normally distributed, and exhibited a positively skewed distribution. In the first and second rounds, the data of the patients who did not undergo consultation for more than 24 and 8 months were removed, respectively. In the third round, the model achieved the best clustering quality of 0.8, and the patients were divided into five categories, based on the timing of their last consultation. Since the frequency at which the AIDS patients collect their medicine from the designated hospital is once every 3 months, the patients were classified as well adherent if their consultation was within the last 3 months, and poorly adherent otherwise. Two important predictor variables were obtained: the recent consultation month and average medical costs per visit. The C5.0 algorithm was used for the model construction and validation, and the accuracy was 100%. Finally, the only important predictor variable for adherence was the “recent consultation month” represented by R, and the node of the adherence prediction model was “R ≤ 3;R > 3”.

The obtained adherence predictor variable was reliable, which once again validates the rationality of providing free drugs to AIDS patients in the study unit once every 3 months during follow-up consultations, instead of the other used options of 2 weeks or 1 month. This variable can be used as an important predictive marker for adherence to guide the patients towards increasing their adherence. It is simpler and has better operability compared with the methods previously used.

Since ART is a lifelong treatment, the AIDS patient services should be expanded and simplified [31]. Here we present the following recommendation in this matter. In China, the actual names are used for mobile phone, telephone and internet users; thus, we recommend that the hospitals construct an information management platform with the following tasks: (1) When the patient’s mobile phone opens the information management platform, a “drug administration mobile phone check in” pop-up automatically appears. A mobile phone SMS or a fixed telephone line call reminds the patient to take their medicines if they did not check in; (2) Every time the patient comes to take the free drugs, the workstation automatically calculates the next date for drug collection and continuously pushes reminding information three working days before the next consultation date [17, 32]. If the patient does not have a fixed consultation schedule, the system automatically provides feedback to the workstation and activates manual telephone calls.

A main limitation of this study is that there is no cross-sectional study for validation. In addition, the data used in this work come from a single center in Shanghai. Expanding the study by including multi-center data will enrich the conclusions.

## Conclusion

This study constructed and validated a prediction model for medication adherence in AIDS patients, using the RFm model and the k-means and C5.0 algorithms. We obtained an adherence predictor variable, which was the recent consultation month. In future studies, in-depth tracking of adherence in AIDS patients in the study unit and collaboration with other designated medical institutions for treating AIDS patients in other cities in China will be conducted.

## Abbreviations

AIDS: Acquired immunodeficiency syndrome
HIV: The human immunodeficiency virus
ART: Active antiretroviral therapy
RFM: Recency, frequency and monetary value model
CRM: Customer relationship management theory
HIS: Hospital information system
CART: Classification and regression tree (CART)
CHAID: Chi-square Automatic Interaction Detector
QUEST: Quick, Unbiased, Efficient, Statistical Tree
